# Research on the Lift-off Effect of Receiving Longitudinal Mode Guided Waves in Pipes Based on the Villari Effect

**DOI:** 10.3390/s16091529

**Published:** 2016-09-20

**Authors:** Jiang Xu, Yong Sun, Jinhai Zhou

**Affiliations:** School of Mechanical Science and Engineering, Huazhong University of Science and Technology, 1037 Luoyu Road, Wuhan 430074, China; sunyong@hust.edu.cn (Y.S.); zhoujinhai@hust.edu.cn (J.Z.)

**Keywords:** lift-off effect, receiving sensor, Villari effect, longitudinal mode, guided wave, pipe

## Abstract

The magnetostrictive guided wave technology as a non-contact measurement can generate and receive guided waves with a large lift-off distance up to tens of millimeters. However, the lift-off distance of the receiving coil would affect the coupling efficiency from the elastic energy to the electromagnetic energy. In the existing magnetomechanical models, the change of the magnetic field in the air gap was ignored since the permeability of the rod is much greater than that of air. The lift-off distance of the receiving coil will not affect the receiving signals based on these models. However, the experimental phenomenon is in contradiction with these models. To solve the contradiction, the lift-off effect of receiving the longitudinal mode guided waves in pipes is investigated based on the Villari effect. A finite element model of receiving longitudinal guided waves in pipes is obtained based on the Villari effect, which takes into account the magnetic field in the pipe wall and the air zone at the same time. The relation between the amplitude of the induced signals and the radius (lift-off distance) of the receiving coil is obtained, which is verified by experiment. The coupling efficiency of the receiver is a monotonic decline with the lift-off distance increasing. The decay rate of the low frequency wave is slower than the high frequency wave. Additionally, the results show that the rate of change of the magnetic flux in the air zone and in the pipe wall is the same order of magnitude, but opposite. However, the experimental results show that the error of the model in the large lift-off distance is obvious due to the diffusion of the magnetic field in the air, especially for the high frequency guided waves.

## 1. Introduction

The guided wave technology based on the magnetostrictive effect is widely applied to test pipes, cables, and so on [1,2,3,4,5,6,7,8,9,10]. In contrast with the piezoelectric guided wave technology, the magnetostrictive technology can generate and receive guided waves with a large lift-off distance up to tens of millimeters. The axially symmetric modes, such as the L(0,1), L(0,2) and T(0,1) modes, are the most widely-used modes for pipes and cables. The longitudinal mode guided waves are generated based on the magnetostriction effect (known as the Joule effect) and received based on the inverse magnetostriction effect or the magnetomechanical effect (known as the Villari effect). The receiving coil is encircled pipes or cables to receive the longitudinal mode guided waves based on Faraday’s law of induction. In field applications, the transducers are installed as close to the pipe wall as possible to reduce the lift-off effect. However, the lift-off distance cannot be so small to be ignored in many conditions, such as the pipe with the fiber-glass coating, which cannot be removed for installing the transducer (often 3–10 mm; removing and repairing the coatings is too expensive to afford) and the bridge cable with high density polyethylene (HDPE) coatings (HDPE often 5–20 mm; the coatings cannot be removed totally). When we use the magnetostrictive guided wave technology to inspect these pipes and cables, the lift-off distance is comparatively large in spite of the very small lift-off distance between the transducer and the coating surface. To inspect the high temperature pipes, the transducer needs to be installed on the heat-insulation course (often 1–10 mm). Additionally, to inspect the heat exchange tube from inside the pipe, the distance between the transducer and the wall is a few millimeters to allow the transducer to be put inside the tube. Therefore, the lift-off effect needs to be studied for analyzing the results. Meanwhile the coupling efficiency between the magnetic energy and the elastic energy will be affected when the receiver is lifted off. We only care about the magnetic field on the pipe surface when generating guided waves. Nevertheless, the conditions of the total cross-section area of the pipe should be considered when receiving guided waves. Additionally, we find that the amplitude of the receiving signals decreases when the receiving coil is loosened in field. The existing model cannot explain this phenomenon.

Many studies were focused on the model of the magnetomechanical effect. The fundamental theory of the magnetomechanical effect, provided by Jiles, Atherton and Sablik, was based on the “law of approach” [11,12,13,14,15]. The change rate of magnetization with the input elastic energy depends on the displacement from the anhysteretic magnetization. An improved model including the Rayleigh law for the magnetomechanical effect was provided by Li [16]. The model had a better description at small stress or sign changing. A coupled magnetomechanical model for the Villari-effect magnetostrictive sensor was provided by Dapino [17,18]. It could be applied to both magnetostrictive sensors and actuators by a nonlinear partial differential equation system. The magnetic field in the ferromagnetic material was concerned in these models. Nevertheless, these models paid little attention to the distribution of the magnetic field in the air zone. There were several models for receiving guided waves based on the inverse magnetostrictive effect. In the 1950s, Williams R.C. developed a one-dimensional model for detecting guided waves based on magnetostrictive delay lines [19]. The model, which only could be used to solve some simple problems, did not take into account the structure of pipes. Kim Y.Y. et al. gave a model of magnetomechanical effects in a rod under bending for the modal testing [20,21,22,23]. To select the desired wave modes, the bias magnet configuration for mode selection was investigated. In their models, the area of the receiving coil was replaced by the area of the rod to calculate the induced voltage. The change of the magnetic field in the air gap was ignored since the permeability of the rod is much greater than that of air. Based on the existing models, the loosened receiving coil will not affect the receiving signals. However, the experimental phenomenon is in contradiction with these models. This phenomenon indicates that the magnetic field in the air zone to the induced voltage could not be ignored. In order to find the cause of the conflict, it is essential to study the distribution of the magnetic field in the pipe cross-section (the pipe wall and the air zone) when the wave passes. On the one hand, it is helpful to understand why the magnetostrictive technology can receive guided waves with a large lift-off distance. On the other hand, it is useful to design and arrange the Villari effect magnetostrictive sensor.

In this paper, we aim to obtain the distribution of the rate of change of the magnetic field in the pipe cross-section to study the relation between the lift-off distance of the receiving coil and the magnetomechanical coupling efficiency for receiving longitudinal guided waves in pipes. A finite element (FE) model considering the changing magnetic field in the pipe wall and the air zone at the same time is obtained. The relation between the amplitude of the induced signals and the radius (lift-off distance) of the receiving coil is obtained, which is verified by experiment. The results show that the coupling efficiency of the receiver is a monotonic decline with the lift-off distance increasing. The decay rate of the low frequency wave is slower than the high frequency wave. Additionally, the results show that the rate of change of the magnetic flux in the air zone and in the pipe wall is the same order of magnitude, but opposite.

## 2. Theory Background

When a ferromagnetic material during magnetization is subjected to the change of a stress, the change of magnetization in the material is exhibited. This phenomenon is defined as the inverse magnetostriction effect or the magnetomechanical effect. The longitudinal inverse magnetostriction effect is called the Villari effect. When the longitudinal mode guided waves propagate in the pipe, the stress in the propagating position is changed. Moreover, the change of the magnetic flux, which can be induced by an encircling coil, is exhibited based on the Villari effect. Under an optimal strength axial bias magnetic field, the Villari effect of the ferromagnetic metal can be linear and non-hysteretic when the induced magnetic field is far less than the bias field. From the principle of energy conservation, the relation of the mechanical and the magnetic properties can be expressed as:

(1) $$S=s^{H}T+dH$$

(2) $$B=eT+\mu^{T}H$$

where *B* is the magnetic flux density induced by the stress, *e* is the magnetostrictive coefficient, *T* is the stress applied to the ferromagnetic material, *μ^T^* is the permeability of the ferromagnetic material at constant *T* and *H* is the strength of the applied magnetic field; where *S* is the strain, *s^H^* is the elastic compliance at constant *H*, *H* is the magnetic field strength, *T* is the stress, *d* is the magnetostrictive cross-coupling coefficient, *B* is the magnetic flux density and *μ^T^* is the permeability at constant *T*. The piezomagnetic equation is normalized in the IEEE Standard 319-1990 [24]. Equation (2) gives the relation between the stress and the magnetic flux density as the Villari effect. In terms of Le Chatelier’s principle, a thermodynamic relation between the Joule effect and the Villari effect could be given:

(3) $${\frac{\partial S}{\partial H})}_{T}={\frac{\partial B}{\partial T})}_{H}$$

where ${\frac{\partial S}{\partial H})}_{T}$ is the rate of change of magnetostriction with the magnetic field at the constant stress and ${\frac{\partial B}{\partial T})}_{H}$ is the change of the magnetic flux density with the stress at the constant field. Equation (3) shows that if the change rate of the magnetostriction is fixed, the change of the magnetic flux density is the same when the receiving coil is open and the bias magnetic field is fixed. Here, the dispersion and the attenuation of the elastic waves are ignored. In other words, if the excitation conditions, such as the excitation position, the excitation frequency and the excitation current, are fixed, the distribution of the magnetic flux density around the pipe at the receiving position is the same at the same moment. Therefore, if we could obtain the distribution of the rate of change of magnetic flux in the pipe cross-section (the pipe wall and the air zone), the amplitude of the induced signal could be calculated by using Faraday’s law of induction.

## 3. FE Model

**Figure 1:**
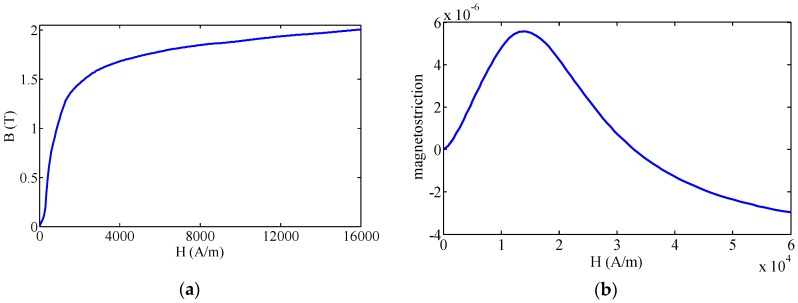
The magnetization curve and the magnetostriction curve of the steel pipe. (**a**) The magnetization curve; (**b**) the magnetostriction curve.

**Figure 2:**
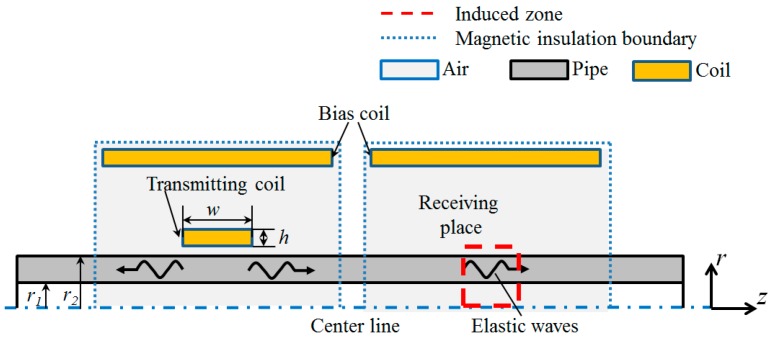
Schematic diagram of the FE model for generating and receiving guided waves based on the magnetostrictive effect.

An FE model based on the Joule effect and the Villari effect is simulated using commercial FE software, COMSOL Multiphysics. This software can solve the magnetic-acoustic coupled problem. Moreover, the custom constitutive equations can be input easily. The magnetization and magnetostriction curves of the steel pipe are displayed in Figure 1. Here, a 2D axisymmetric FE model is built up as we focus only on the axisymmetric mode waves. Based on the custom constitutive equations mentioned above, a 2D axisymmetric FE model is built up, which contains both the transmitting and receiving process. In the FE model, the AC/DC module is used to simulate the magnetic field, while the solid mechanics module is applied in the sound field calculation. Due to the skin layer being the region where the magnetic field changes fiercely, the inner and outer skin layer are set in the simulation model. The area of inner and outer skin layers (0.03 mm depth and 80 mm length) are mapped in a square grid; the other adjacent areas (air, tube, etc.) are free triangular meshed. Two skin layers are divided into 10 layers in the depth direction and 80 parts in the length direction; adjacent areas are free triangular meshed with the minimum size of 0.036 mm and the maximum size of 0.8 mm. The schematic diagram of the FE model is shown in Figure 2, and the parameters of the FE model used in this work are shown in Table 1. Firstly, the bias magnetic field induced by the bias coil at the excitation place and the receiving place is calculated. The permeability and the magnetostriction matrix is obtained. Secondly, a four-cycle Hanning windowed tone burst is employed on the transmitting coil. The dynamic magnetic field produced by the transmitting coil is calculated. Thirdly, the elastic wave generated in the pipes based on the Joule effect is calculated. Magnetostrictive strain is calculated through Equation (1) based on the dynamic magnetic field strength and the magnetostriction matrix. Elastic waves generated by the strain are simulated through an elastic analysis. The displacement vector, stress and strain of a particle induced by elastic waves in the pipe are calculated by taking the second category Piola–Kirchhoff stress into consideration. Fourthly, the dynamic magnetic field in the induced zone at the receiving place is calculated. The magnetic flux density in the pipe wall is calculated through Equation (2) based on the permeability of the pipe and the magnetic field strength of the pipe. The magnetic flux density in the air zone is calculated by using the magnetic field strength of the air and the permeability of the air. The rate of change of the magnetic flux density in the radial direction is obtained by differential computing. Finally, Equation (4) is used to calculate the change rate of the magnetic flux waveforms from every ring (the area of each ring is different because the size of each grid is different), Equation (5) to calculate the induced electromotive force waveforms at different radial position and extracting the peak-to-peak value from the induced electromotive force waveforms.

(4) $$d\varphi_{r_{m}}/dt=dB_{z}(r_{m})/dt\times \pi ({r_{m}}^{2}-{r_{m-1}}^{2})$$

(5) $$U(r_{m})=nd\varphi (r_{m})/dt=n\sum_{k=1}^{m}d\varphi_{r_{k}}/dt$$

**Figure 3:**
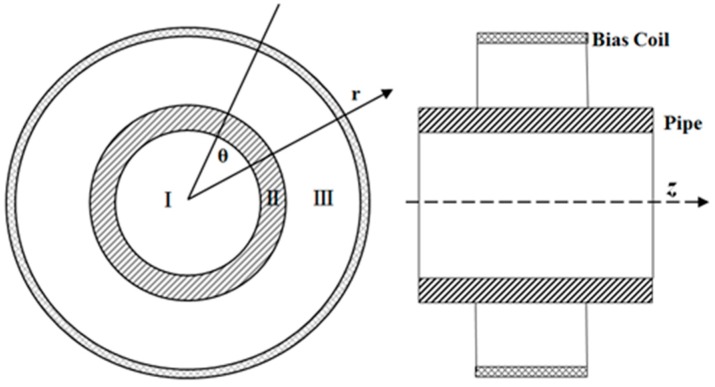
Layout of the model at the receiving position.

To calculate the induced electromotive force of the receiving coil, we define three zones in the radial direction of the receiving place, as shown in Figure 3. Zone I is the air zone internal the pipe. Zone II is the pipe wall. Zone III is the air zone from the external surface of the pipe to the bias coil.

From Faraday’s law of induction and the z-axial symmetry of the magnetic field, the induced electromotive force of the receiver coil is calculated in two conditions.

case 1 the receiving coil in Zone I:
  (6) $$\varphi_{in}(r_{in},t)=\varphi_{I_{1}}(t)$$
  (7) $$V(r_{in},t)=-\frac{nd\varphi_{in}}{dt}=-n\frac{d}{dt}\underset{I_{1}}{\int }B_{I_{1}}dS_{I_{1}}=-2\pi n\frac{d}{dt}\int_{0}^{r_{in}}B_{I}(r,t)dr$$
case 2 the receiving coil in Zone III:
  (8) $$\varphi_{out}(r_{out},t)=\varphi_{I}(t)+\varphi_{II}(t)+\varphi_{III_{1}}(r_{out},t)$$
  (9) $$\begin{array}{l} V(r_{out},t)=-\frac{nd\varphi_{out}}{dt}=-n\frac{d}{dt}(\underset{I}{\int }B_{I}dS_{I}+\underset{II}{\int }B_{II}dS_{II}+\underset{III_{1}}{\int }B_{III_{1}}dS_{III_{1}})\\ =-2\pi n\frac{d}{dt}(\int_{0}^{r_{1}}B_{I}(r,t)dr+\int_{r_{1}}^{r_{2}}B_{II}(r,t)dr+\int_{r_{2}}^{r_{out}}B_{III_{1}}(r,t)dr) \end{array}$$

## 4. Experimental Setup

**Figure 4:**
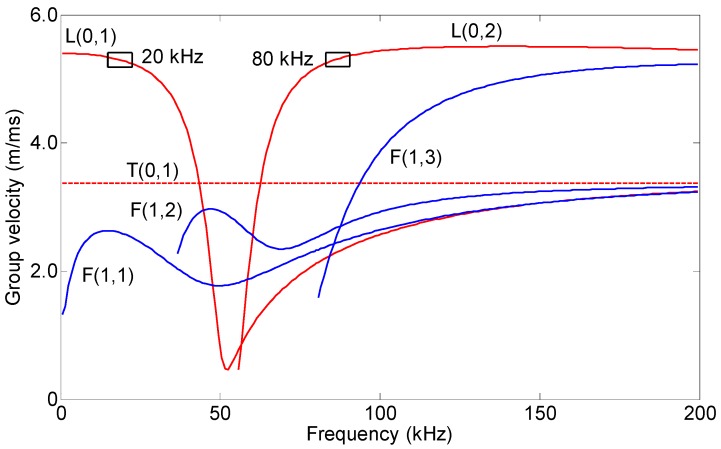
Group velocity dispersion curve of the steel pipe with a 38 mm outer diameter and a 5 mm wall thickness.

A steel pipe, with a 38 mm outer diameter, a 5 mm wall thickness and 3.2 m long, was employed as the specimen. The group velocity disperse curve of the pipe is shown in Figure 4, which was calculated by DISPERSE software [25]. The transmitting coil and the receiving coils were placed at approximately 1 m and 0.6 m from the near end of the pipe, respectively. The bias coils on the excitation side and on the receiving side were the same, which were made of No. 18 gauge wire, with 500 turns, 110 mm in length and 160 mm in diameter. The transmitting coil was made of No. 24 gauge wire, with 20 turns, an 11 mm length and a 38.5 mm inner diameter. The transmitting coil and the bias coils encircled the pipe from the outside. The receiving coils were made of No. 24 gauge wire, with 20 turns, 11 mm in length. There were two group coils. The first group coils were placed in the internal air of the pipe, and the second group coils were placed in the external air of the pipe. The diameters of the first group coils were 13 mm, 17 mm, 21 mm, 24.7 mm and 26.2 mm. The diameters of the second group coils were 38.5 mm, 42 mm, 50 mm, 57 mm, 64 mm, 71 mm, 77 mm, 84 mm, 92 mm, 100 mm, 110 mm, 116 mm, 122 mm, 130 mm, 138 mm and 144 mm. The amplitudes of the bias current at the excitation side and the receiving side were 3 A. Because the dispersion is harmful to the testing, the excitation frequency of the mode with little dispersion is preferred. The excitation frequencies were 20 kHz (L(0,1)) and 80 kHz (L(0,2)). The excitation signal was a four-cycle sinusoidal tone burst with a Hanning window. The peak value of the excitation current was 5 A. The induced signals by the receiving coils were amplified by approximately 66 dB. The signals were subsequently digitized using an A/D card, which works at 2 M sample per second. The repeated times were 300 times to reduce the white noises.

## 5. Results and Discussions

**Figure 5:**
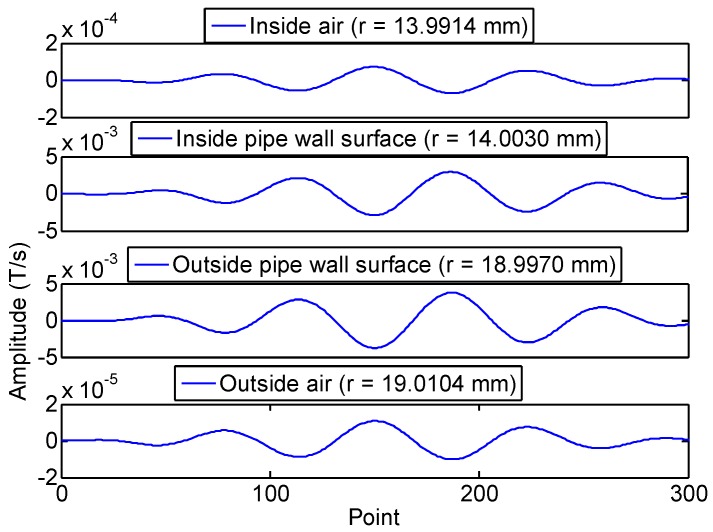
The FE calculation results of the changing rate of magnetic flux density of four radial points at 20 kHz.

**Figure 6:**
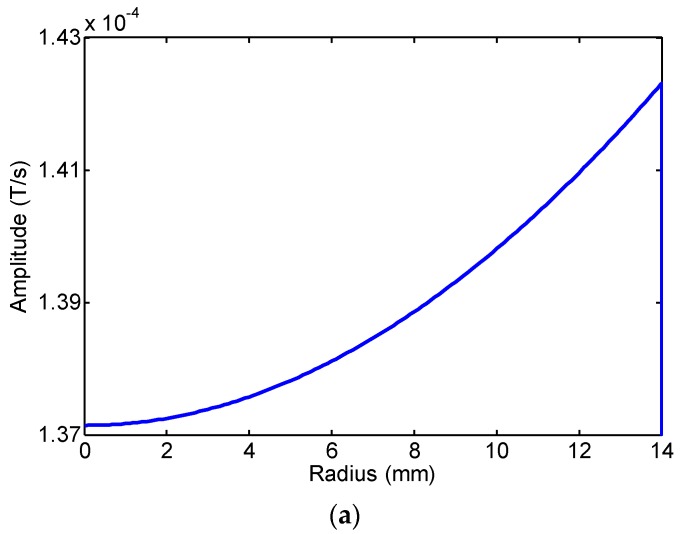

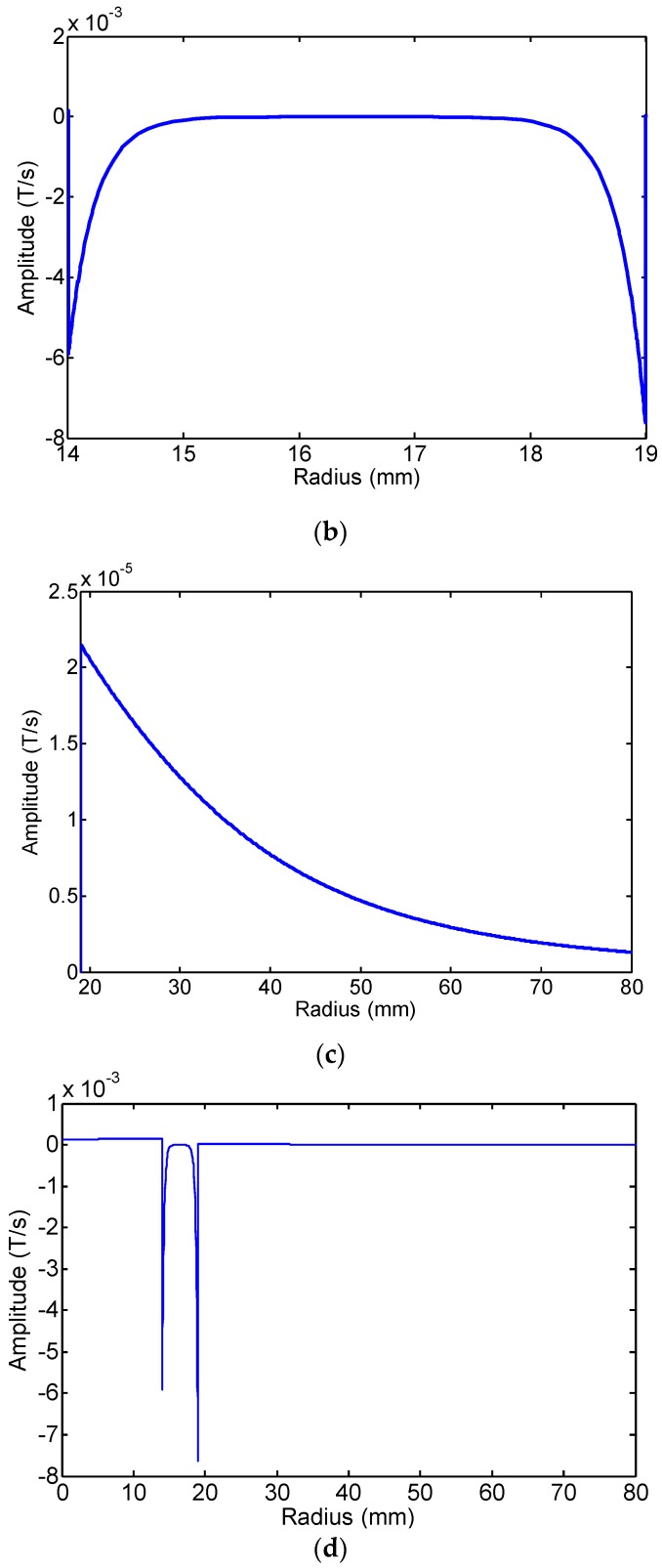
The FE calculation results of the changing rate of magnetic flux density in the radial direction at 20 kHz: (**a**) the internal air zone; (**b**) the pipe wall; (**c**) the external air zone; (**d**) the 0–80 mm zone.

**Figure 7:**
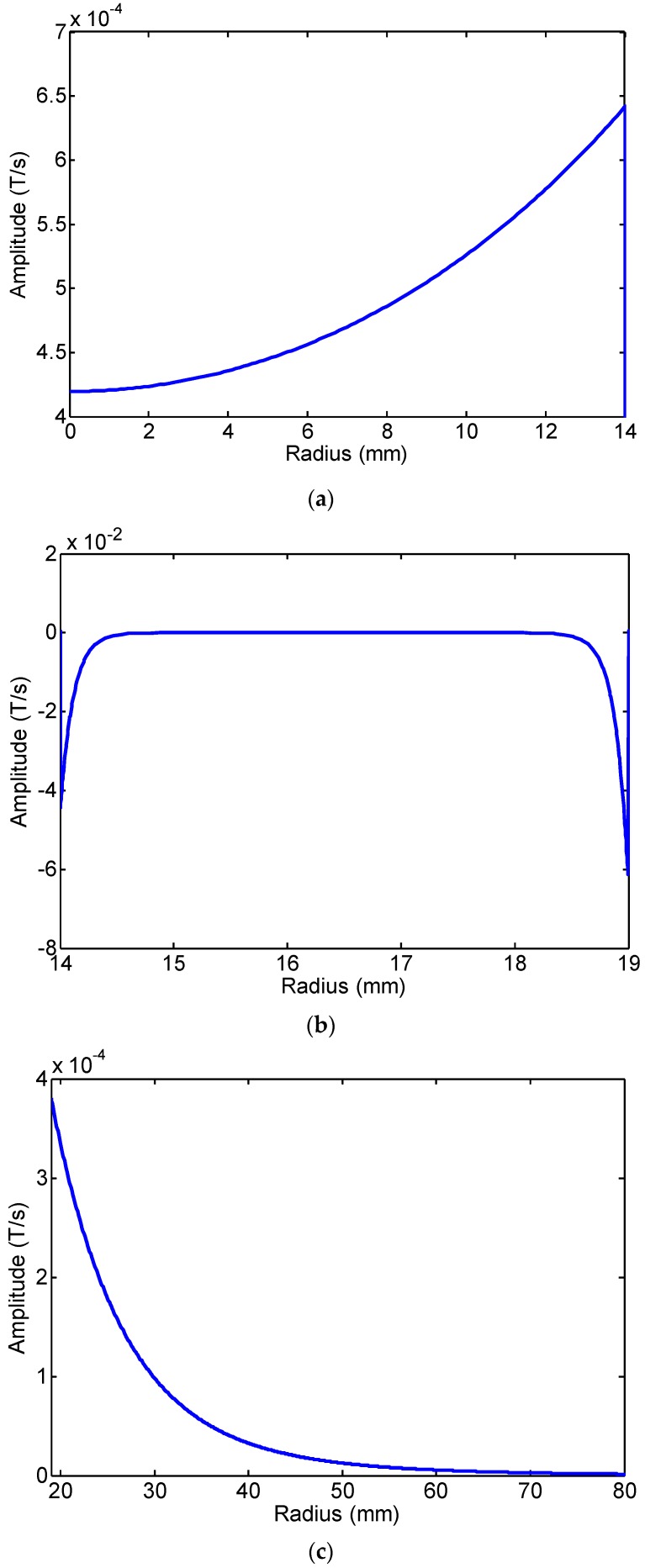

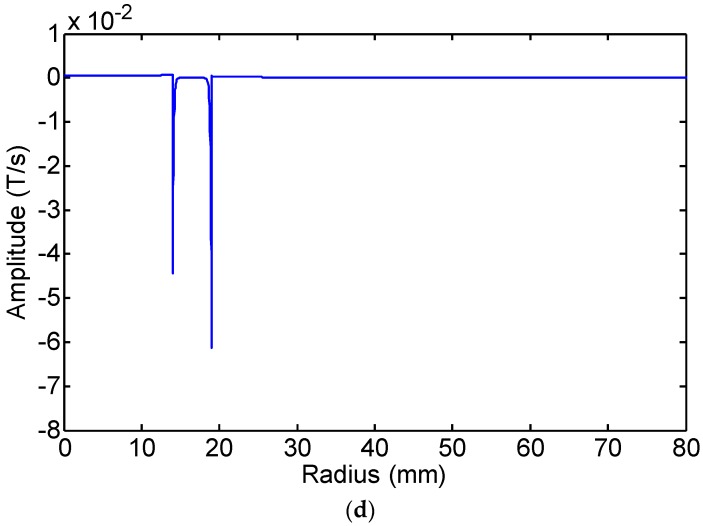
The FE calculation results of the changing rate of magnetic flux density in the radial direction at 80 kHz: (**a**) the internal air zone; (**b**) the pipe wall; (**c**) the external air zone; (**d**) the 0–80 mm zone.

The FE calculation results of the changing rate of magnetic flux density of four radial points (internal air zone, pipe internal surface, pipe external surface and external air zone) of the first passing signal at 20 kHz are shown in Figure 5. The rate of change of the magnetic flux density in the pipe wall and in the air zone is inverted. Here, we define that the changing rate of magnetic flux density is positive in the air zone and negative in the pipe wall. The FE calculation peak-peak value of the changing rate of magnetic flux density in the radial direction at 20 kHz and 80 kHz is shown in Figure 6 and Figure 7. The changing rate of magnetic flux density distribution in Zone I (the internal air zone) and Zone III (the external air zone) are in the same direction, but the opposite direction in Zone II (the pipe wall). The changing rate of magnetic flux density distribution in Zone I is relatively uniform. The changing rate of magnetic flux density in Zone II is concentrated on the internal and external surfaces by the skin effect. The decay speed of 80 kHz is quicker than 20 kHz with greater depths in the pipe wall. The maximum value of the rate of change of the magnetic flux density in the pipe wall is almost two orders of magnitude larger than that of the air zone at 20 kHz and 80 kHz. The reason is that the permeability of the steel pipe is much greater than that of air. However, the induced electromotive force depends on the rate of change of the magnetic flux enclosed by the receiving coil. Therefore, the rate of change of the magnetic flux in the air zone should not be ignored based on this reason.

**Figure 8:**
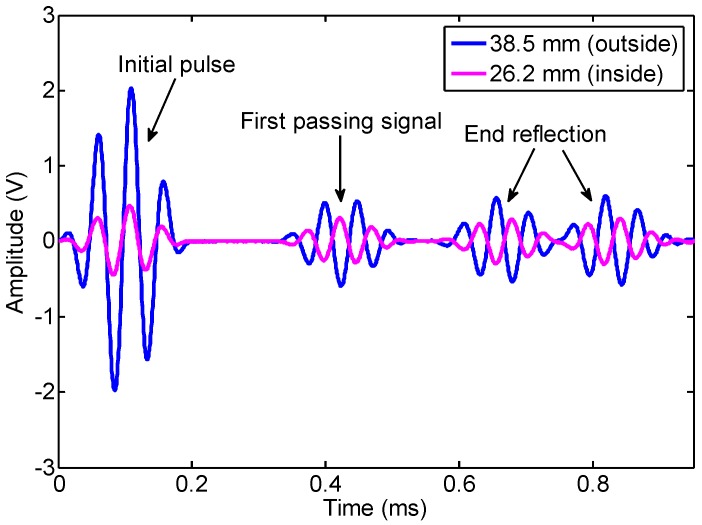
The signals induced by the internal coil with a 26.2 mm diameter and the external coil with a 38.5 mm diameter at 20 kHz.

**Figure 9:**
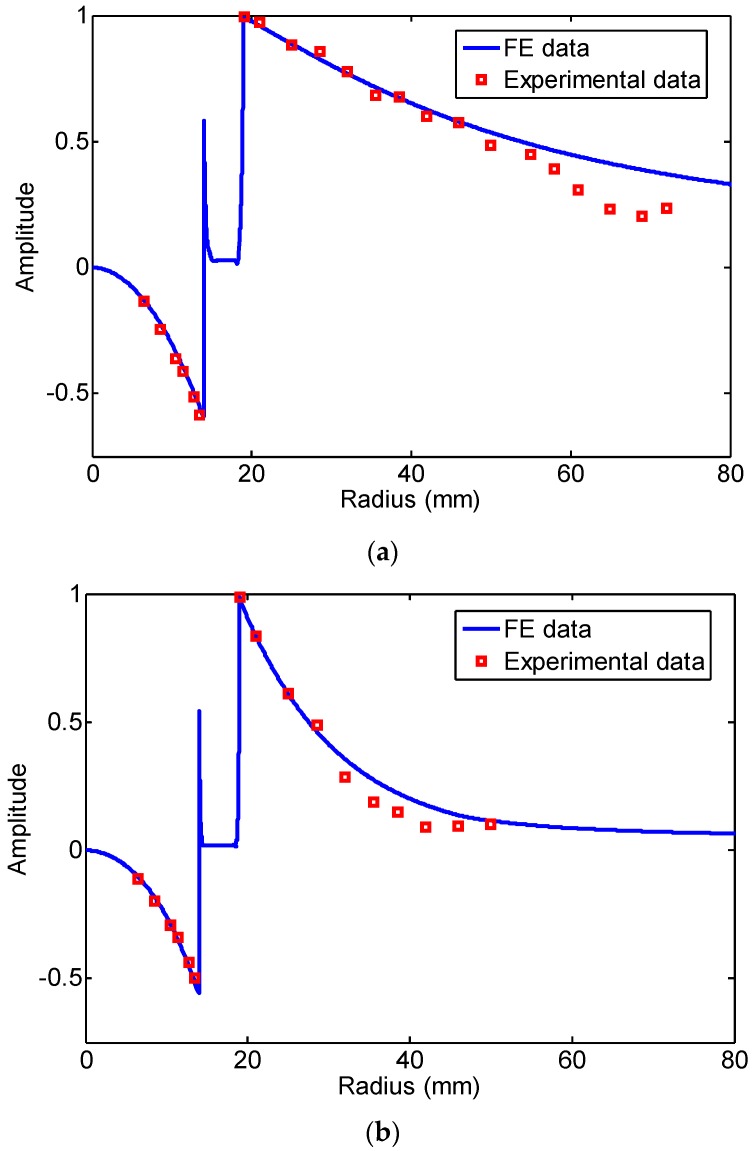
The normalized experimental and FE data of the peak-peak value of the first passing signal: (**a**) 20 kHz; (**b**) 80 kHz.

In the following, we will discuss the effect of the changing rate of magnetic flux in the air zone on the induced signal. First, the experimental signals from the internal coil with a 26.2 mm diameter and the external coil with a 38.5 mm diameter at 20 kHz are shown in Figure 8. The two signals of the first passing signal are inverted. Here, the peak-peak value of the first passing signal by the external coil is defined as positive and by the internal coil as negative, which is in accordance with the definition of the FE results. The normalized experimental and FE data of the peak-peak value of the first passing signal at 20 kHz and 80 kHz are shown in Figure 9. The FE simulation results were normalized as the peak-to-peak value of induced signal when the receiving coil was placed at the outside pipe with 0 mm lift-off. The reasons for the difference between the FE data shown in Figure 6, Figure 7 and Figure 9 are:

a) The magnetic flux density in the pipe wall is concentrated on the internal and external surfaces by the skin effect. The maximum value of the change rate of magnetic flux density in the pipe wall surface is almost two orders of magnitude larger than that of the air zone. The reason is that the permeability of the steel pipe is much greater than that of air.
b) The effective area in the pipe wall is very limited, and the magnetic flux density in the pipe wall is decreased quickly due to the skin effect. The magnetic flux density in the pipe wall is very small (*dB/dt* = −1.686 × 10^−6^ T/s, *r* = 16.5 mm, *f* = 80 kHz). Although the magnetic flux density in the air zone is less than two orders of magnitude that in the pipe wall, the decay rate of magnetic flux density in the air zone is less than that in the pipe wall. Additionally, the effective area in the air zone is larger than that in the pipe wall.

The induced electromotive force depends on the change rate of the magnetic flux density and the effective area enclosed by the receiving coil. Overall, although the maximum value of the change rate of magnetic flux density in the pipe wall surface is almost two orders of magnitude larger than that of the air zone, the induced electromotive force in the air zone and in the pipe wall is in the same order.

The experimental results were in agreement with the FE data at small radius or small lift-off distance (20 kHz less than 57 mm radius, 80 kHz less than 28.5 mm radius). When the lift-off distance of the receiving coil was large, the experimental results were less than the FE data. The results were in accord with the curve given by Thompson R.B. [26]. The reason was that the magnetic field was diffused in the air zone far from the pipe wall, which led to the weak signals and the stronger noise. The phenomenon was much more obvious when the higher frequency was applied.

Here, if we supposed that the amplitude of the signal induced in the pipe wall was 100%, the amplitude of the signal induced in Zone I was about 36.7%. The amplitude of the signal induced in Zone III could reach 63.3% if the area of Zone III is large enough. When the radius of Zone III is from 19 mm–50 mm, the amplitude of the induced signal was about 32% at 20 kHz and 57% at 80 kHz. The rate of change of the magnetic flux in the air zone and in the pipe wall was in the same order of magnitude. Therefore, the rate of change of the magnetic flux in the air zone should not be ignored. Based on this model, the lift-off distance of the receiving coil should be as small as possible in application. By only considering the guided wave receiving based on the Villari effect, the low frequency waves are more applicable to test pipes with a large lift-off distance than the high frequency waves. Additionally, we can receive the longitudinal mode guided waves in pipes by only inducing the rate of change of the magnetic flux in the air zone, as our former paper mentioned [27].

## 6. Conclusions

The lift-off effect of receiving the longitudinal mode guided waves in pipes is investigated based on the Villari effect in this paper. A model of receiving longitudinal guided waves in pipes is obtained based on the Villari effect, which takes into account the magnetic field in the pipe wall and the air zone at the same time. The changing rate of magnetic flux density in the pipe wall and in the air zone is inverted. The magnetic flux density in the pipe wall is concentrated on the internal and external surfaces by the skin effect. The maximum value of the change rate of magnetic flux density in the pipe wall surface is almost two orders of magnitude larger than that of the air zone. The reason is that the permeability of the steel pipe is much greater than that of air. However, the induced electromotive force depends on the change rate of the magnetic flux enclosed by the receiving coil. The change rate of the magnetic flux in the air zone and in the pipe wall is in the same order of magnitude, but opposite. If we supposed that the amplitude of the signal induced in the pipe wall is 100%, the amplitude of the signal induced in internal air zone can reach up to 30%. The amplitude of the signal induced in the outer air zone can reach up to 60%. Therefore, the rate of change of the magnetic flux in the air zone should not be ignored. Due to the negative contribution of the air zone to the induced signal, the amplitude of the receiving signal will decrease with the lift-off distance increasing. The relationship between the amplitude of the receiving signal and the lift-off distance of the receiving coil is obtained, which is verified by experiments. The results will provide support to inspecting pipes with a lift-off distance based on the magnetostrictive effect. Additionally, the model is not applicable when the lift-off distance is large due to the diffusion of the magnetic field in air, especially for the high frequency guided waves. We will modify the model by considering the diffusion of the magnetic field when the lift-off distance is large in the future work.

## Figures and Tables

**Table 1:** Parameters of the FE model used in this work.

Object	Parameters	Symbol	Value
Pipe	Inner radius	*r*_1_	14 mm
	Outer radius	*r*_2_	19 mm
	Conductivity	*σ*	1.12 × 10^7^ S/m
	Youngs Modulus	*E*	210 GPa
	Poissons ratio	*ν*	0.29
	Density	*ρ*	7870 kg/m^3^
Bias coil	Width	*w_b_*	110 mm
	Height	*h_b_*	5 mm
	Inside diameter	*ID_b_*	160 mm
	Turns number	*N_b_*	500
	Conductivity	*σ*	6.7 × 10^7^ S/m
	Permeability	*u_rc_*	1
Excitation coil	Width	*w_T_*	11 mm
	Height	*h_T_*	0.5 mm
	Liftoff	*lf*	0.25 mm
	Turns number	*N_T_*	20
	Conductivity	*σ*	6.7 × 10^7^ S/m
	Permeability	*u_rc_*	1
Excitation parameters	Amplitude	*I*	5 A
	Center frequency	*f*	20 kHz, 80 kHz
Receiving coil	Width	*w_R_*	11 mm
	Height	*h_R_*	0.5 mm
	Turns number	*N_R_*	20
